# Intravenous Thrombolysis for Ischemic Stroke Patients with Pituitary Neoplasms: A Nationwide Study and Scoping Review

**DOI:** 10.3390/neurosci7010019

**Published:** 2026-02-02

**Authors:** Matthew K. McIntyre, Huanwen Chen, Dheeraj Gandhi, Ajay Malhotra, Ryan Priest, Marco Colasurdo

**Affiliations:** 1 Department of Neurological Surgery, Oregon Health & Science University, Portland, OR 97239, USA; 2 Department of Neurology, MedStar Georgetown University Hospital, Washington, DC 20007, USA; 3 Department of Neurosurgery, University of Maryland Medical Center, Baltimore, MD 21201, USA; 4 Department of Radiology, Yale New Haven Hospital, New Haven, CT 06510, USA; 5 Department of Interventional Radiology, Oregon Health & Science University, Portland, OR 97239, USA

**Keywords:** stroke, pituitary neoplasm, intravenous thrombolysis, TPA, TNK, hemorrhage

## Abstract

Objective: The safety of intravenous thrombolysis (IVT) for acute ischemic stroke (AIS) patients with pituitary neoplasms is unclear. This study aims to assess IVT’s safety and efficacy in this patient population. Methods: We reviewed PubMed, Scopus, EMBASE, and Web of Science through July 2025 for reports of IVT administration in AIS patients with pituitary neoplasia. We also performed a retrospective analysis of the Nationwide Readmissions Database (NRD) from 2016 to 2022 to compare outcomes of IVT versus no IVT for AIS patients with pituitary neoplasia, and outcomes of IVT-treated AIS patients with versus without pituitary neoplasia. Outcomes of interest include post-stroke functional status, intracranial hemorrhage (ICH), mortality, and pituitary apoplexy. Multivariate regression analyses were performed to adjust for confounders. Results: The literature review identified 5 AIS patients with pituitary neoplasia, of whom 3/5 (60%) experienced intracranial hemorrhage and none developed apoplexy. In the nationwide analysis of 1,246,750 AIS patients, 1661 (0.13%) had concomitant pituitary neoplasm. Among these patients, IVT was associated with higher odds of functional independence at discharge (adjusted OR 2.46 [95%CI 1.56–3.87]), without increased risk of ICH or in-hospital death (*p* > 0.05). No cases of pituitary apoplexy were observed. Outcomes among all IVT-treated AIS patients did not differ between those with and without pituitary neoplasms (all *p* > 0.05). Interpretation: Only five cases of IVT for AIS patients with pituitary neoplasia were identified, highlighting a striking lack of clinical data. In a large U.S. cohort of AIS patients, IVT was associated with improved hospitalization outcomes without increased risk of ICH or pituitary apoplexy.

## 1. Introduction

Intravenous thrombolysis (IVT) remains a mainstay in the treatment of acute ischemic stroke [1,2]. The use of intravenous thrombolysis in patients presenting with an acute ischemic stroke (AIS) with a known intracranial neoplasm is currently contraindicated on the FDA labeling of both alteplase and tenecteplase. Current American Heart Association (AHA) guidelines state that for AIS, IVT is probably recommended in those with extra-axial intracranial neoplasms and potentially harmful in those with intra-axial neoplasms [3]. While considered intracranial and extra-axial neoplasms, pituitary lesions are often deemed distinct, given the elaborate vascular tree that encircles the gland and the unique risk of spontaneous apoplexy [4].

Like extra-axial neoplasms, such as meningiomas, the pituitary gland receives arterial supply from both intradural (superior hypophyseal) and extradural (inferior hypophyseal) arteries to supply the anterior and posterior pituitary gland, respectively [5]. Unlike meningiomas, however, during a hemorrhage event, the pituitary gland’s position below the diaphragma sellae results in the unique phenomenon of pituitary apoplexy rather than classical intracranial hemorrhage. Moreover, while most pituitary adenomas are non-functional, certain functional adenomas, such as those in Cushing’s disease, may result in a markedly pro-thrombotic state that results in a near doubling of a patient’s stroke risk [6]. These effects, and the systemic effects of endocrinopathy because of a pituitary adenoma, make these lesions distinct from other extra- and intracranial neoplasia.

Data on acute apoplexy or intracranial hemorrhage (ICH) after IVT for acute ischemic stroke are scarce, leaving providers with limited evidence to support the decision for or against IVT in this patient population. In this study, we aimed to investigate the safety of IVT in AIS patients with pituitary or sellar neoplasia through a dual study design. First, we attempted to systematically evaluate the literature to evaluate for prior work on the safety of IVT in AIS patients with pituitary or sellar neoplasia. Second, given the paucity of cases identified in the literature review, we go on to perform a large nationwide study evaluating the incidence of ICH, mortality, and discharge outcomes in patients with AIS and concurrent pituitary neoplasm. We hypothesized that those with a pituitary neoplasm receiving IVT are not at increased risk for adverse events compared to the general population.

## 2. Methods

### 2.1. Scoping Review Methods

PubMed, EMBASE, Scopus, and Web of Science databases were searched from inception until July 2025 for English-language clinical articles evaluating the use of IVT in patients with AIS and pituitary or sellar neoplasia. The search strategies for each database are detailed in Table S1 and included all original articles, letters, and conference abstracts (including case reports, case series, cohort studies, and clinical trials), but excluded review articles and duplicates. Titles and abstracts were independently screened by two independent study investigators for eligibility, followed by full-text screening by two independent study investigators. We designed our search strategy with the goal of performing a systematic review; however, a scoping review was performed, given the severe lack of published literature identified.

### 2.2. National Readmissions Database Study Design

We also conducted a retrospective analysis of the 2016 to 2022 Nationwide Readmissions Database (NRD), which is part of the Healthcare Cost and Utilization Project (HCUP). The NRD provides information on all hospitalization records of admitted and readmitted patients across 30 geographically dispersed states. Patient identifiers are not included in the NRD; as such, this study was exempt from institutional review board approval.

### 2.3. Patient Identification and Inclusion in the NRD

Patients who were hospitalized non-electively for AIS were identified using validated ICD-10 codes [7,8,9]. Patients were excluded if they had an asymptomatic stroke (NIH stroke scale of 0 or missing); underwent endovascular therapy or non-acute IVT administration; had another neoplasm, endocarditis, vasculitis, cerebral amyloid angiopathy, moyamoya disease, or an intracranial vascular malformation [10]; had missing data; or had primary residence in a different state. Patients were then stratified into those with a pituitary mass and those without.

### 2.4. Variables of Interest and Endpoints in the NRD

Age, sex, teaching hospital status, initial NIH stroke scale, anticoagulant and antiplatelet medication use, and comorbidities in the Elixhauser comorbidity index were identified. Primary endpoints were ICH, routine discharge, home discharge, and in-hospital mortality. Routine discharge is defined in the US Medicare discharge disposition codes as a discharge to home without the need for additional services, while home discharge is a discharge to home with the need for additional services such as physical therapy or durable medical equipment. The incidence of pituitary apoplexy was also evaluated. All ICD-10 codes used in this study are presented in Table S2.

### 2.5. Statistical Methods for NRD Analysis

First, we estimated the sample size needed for our study. Assuming that 30% of untreated stroke patients with pituitary tumors are discharged without inpatient or home rehabilitation needs, a sample size of 93 IVT-treated patients is needed to detect a 20% rate difference with IVT treatment. Continuous variables were reported as median and interquartile range, and categorical variables were reported as percentages. Patient characteristics and discharge outcomes were compared between patient groups using non-parametric tests or chi-squared tests. Discharge outcomes were also compared with additional multivariable adjustments using either binary regression models for dichotomous endpoints. Covariables used for adjustments were chosen a priori, and included age, sex, stroke severity (NIH stroke scale), hypoxemia, anemia, hypertension, hyperlipidemia, diabetes, smoking history, congestive heart failure, ischemic heart disease, peripheral artery disease, deep vein thrombosis, pulmonary embolism, chronic antithrombotic medication use, chronic kidney disease, liver disease, obesity, hospital type, and treatment year. These variables were all chosen based on their known influence on stroke severity and outcome. Variance Inflation Factor (VIF) was calculated for all covariates to assess for collinearity, with VIF values < 5 deemed acceptable. Per HCUP guidelines, values less than 10 are not reported. *p*-values less than 0.05 were deemed statistically significant. All statistical analyses were performed using R, Version 3.6.2 (https://www.r-project.org/).

## 3. Results

### 3.1. Scoping Review

**Figure 1:**
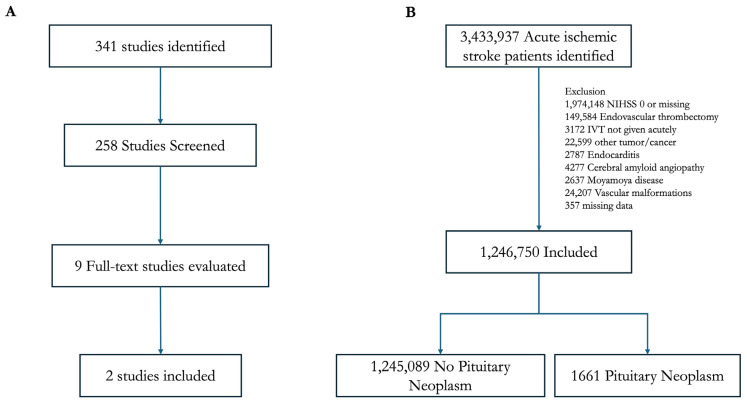
Inclusion and exclusion criteria for the scoping review (**A**) and national readmission database (**B**) portions of the study.

The scoping review screening identified 341 studies, of which 218 were from Web of Science, 50 Scopus, 40 PubMed, and 33 Embase. After screening and removing duplicates, nine studies underwent full-text evaluation for eligibility, of which two met the inclusion criteria (Figure 1A). Five patients with pituitary or sellar neoplasia with AIS who subsequently received IVT were identified. Of these patients, 3/5 (60%) experienced intracranial hemorrhage, 2/5 (40%) experienced symptomatic ICH, and none experienced apoplexy following IVT (Table 1). Given the small sample size available, meta-analysis was not performed.

### 3.2. NRD Patient Characteristics

Of the 3,433,937 patients with AIS, 1,246,750 met the inclusion and exclusion criteria, of whom 1661 (0.13%) were known to have a concomitant pituitary neoplasm (Figure 1B). Those with a pituitary neoplasm had a median age of 72 (IQR: 60–81) and a slight male predominance (56.3%) (Table 2). Compared to those without an underlying lesion, patients with a pituitary neoplasm were more likely to be male (*p* = 0.003), be at a teaching hospital (*p* = 0.01), less likely to be taking anti-coagulation (*p* < 0.001), and had a lower comorbidity burden (*p* < 0.001). Both groups had a mean NIH stroke scale score of 4, which was statistically significant between groups but not meaningfully different clinically. Those with pituitary neoplasms were significantly less likely to receive IVT (14.4% v. 17.3%, *p* = 0.017) compared to those without pituitary lesions prior to and after adjusting for covariables (adjusted odds ratio (aOR) 0.81 [95%CI: 0.68–0.97], *p* = 0.022).

### 3.3. NRD Outcomes

Among patients with known pituitary neoplasia, 240 patients received IVT, satisfying pre-determined statistical power requirements to detect a meaningful difference in routine discharge compared to no IVT treatment. IVT was associated with significantly increased odds of routine discharge (aOR 2.46 [95%CI: 1.56–3.87], *p* < 0.001) and home discharge (aOR 2.63 [95%CI: 155–4.45], *p* < 0.001), without associated differences in ICH (aOR 0.77 [95%CI: 0.35–1.72], *p* = 0.53) or mortality (aOR 1.72 [95%CI: 0.49–6.04], *p* = 0.40; Table 3). There were no cases of pituitary apoplexy among patients with pituitary neoplasm treated with IVT. VIFs for all covariates within multivariable models were less than 5, indicating no substantial collinearity. Among IVT-treated patients, interaction analysis demonstrates that there were no significant differences in any of the examined outcomes between those with and without pituitary neoplasms (all interaction *p* > 0.05; Table 2), with a slight trend toward decreased ICH events in patients with pituitary neoplasms (OR 0.38 [95%CI: 0.15–1.02], *p* = 0.054).

## 4. Discussion

In this scoping review and nationwide cohort study, we found that the current literature on stroke thrombolysis for patients with concomitant pituitary neoplasm is extremely limited. In a nationwide cohort of AIS patients in the United States, IVT appeared equally safe and effective for AIS patients with versus without pituitary neoplasms. These findings provide confirmatory evidence in support of current AHA guidelines that generally do not contraindicate administration of IVT for AIS with intracranial, extra-axial masses. Overall, our data provides Class III evidence in support of IVT use among AIS patients with concomitant pituitary neoplasia.

This scoping review of the literature performed in our study on the safety of IVT in AIS patients with pituitary neoplasia revealed a striking shortage of evidence supporting the use of this therapy, with only five cases in the literature, and none of whom developed apoplexy. To date, the largest study examining the safety of IVT in those with pituitary lesions was by Seystahl et al., who found that three of four patients examined developed intracranial hemorrhage after IVT, one of which was fatal, and none were intra-tumoral [11]. One patient had a tumor between 1 and 10 cm^2^, and one patient had a pituitary tumor > 10 cm^2^. That study also included 82 patients with meningioma and 18 with intra-axial tumors, who experienced symptomatic intracranial hemorrhage at a rate of 7% and 6%, respectively. As a result, Seystahl et al. concluded that IVT should be used cautiously in patients with pituitary neoplasia, given the significantly elevated rate of ICH compared to other patients with neoplasia. This study was limited by a lack of comparison to patients without any intracranial neoplasia and a severe lack of statistical power to draw any definitive conclusions with a sample size of only four. The other study identified in our scoping review was by Evans et al., who published a case report of a patient with a pituitary macroadenoma who received IVT for an acute ischemic stroke without subsequent pituitary apoplexy or ICH [12]. While we report that IVT in patients with AIS and pituitary neoplasia experience an intracranial hemorrhage rate of 60% in the literature, this figure represents data from an extremely small sample size with significant publication bias. This bias likely stems from cases of the unusual circumstances surrounding patients with concomitant pituitary neoplasia and should be interpreted very cautiously, especially given the results of our NRD analysis discussed below.

Given the lack of prior evidence on the safety and efficacy of IVT in AIS patients with pituitary neoplasia, we also performed a nationwide retrospective study, finding that IVT provides similar therapeutic benefits, in the form of favorable discharge outcomes, compared to patients without pituitary neoplasia. These benefits come without increased risk of ICH or mortality despite the presence of a pituitary neoplasm. In interaction analysis, IVT was associated with increased odds of favorable discharge location regardless of pituitary neoplasm status. To date, this study is the only cohort study that assesses the safety of IVT in patients with AIS and concomitant pituitary neoplasms.

The feared complication following IVT with either tissue plasminogen activator (TPA) or tenecteplase (TNK) in the treatment of acute ischemic stroke is symptomatic intracranial hemorrhage. In a recent meta-analysis of randomized trials, Zhou et al. compared endovascular thrombectomy (EVT) alone versus endovascular thrombectomy plus IVT for anterior circulation large vessel occlusions, finding that the rate of any intracranial hemorrhage was 36% in those in the IVT group versus 32% in the endovascular therapy alone group. However, the rate of parenchymal hematoma was only 7% in the thrombolysis group versus 4% in the EVT group [13]. Overall, the risks of hemorrhagic conversion after IVT were summarized in a meta-analysis by Sun et al., and included atrial fibrillation, National Institutes of Health stroke score, hyperdense artery sign, number of thrombectomy passes, age, serum glucose, and onset-to-treatment time [14]. While the use of IVT is associated with higher rates of intracranial hemorrhage, there is a net benefit in terms of successful recanalization and overall improvement in functional status [1,2,15].

Current American Heart Association (AHA) guidelines for acute ischemic stroke state that IVT is likely recommended in those with extra-axial intracranial neoplasms and potentially harmful in those with intra-axial neoplasms [3]. At present, the AHA guidelines do not specifically give guidance on patients with known or newly discovered pituitary neoplasia. Likely because of this, our study found that the presence of a pituitary neoplasm was associated with significantly lower odds of IVT utilization among AIS patients compared to controls without pituitary neoplasia. This finding suggests that, despite the presumed safety of IVT among patients with intracranial, extra-axial neoplasms, providers likely remain somewhat reticent about IVT use, particularly for patients with pituitary neoplasms for whom there is very limited data on IVT safety and efficacy. Overall, within the limits of our observational study, our findings are in line with current AHA recommendations.

Beyond intracranial hemorrhage related to the distribution of the stroke, a specific concern in AIS patients with a pituitary neoplasia is the risk of pituitary apoplexy. Pituitary apoplexy is a syndrome characterized by sudden onset headache, vision impairment, vomiting, and potentially endocrinopathy associated with the sudden sellar hemorrhage associated with a pituitary neoplasm [4,16]. The incidence of pituitary neoplasms progressing to pituitary apoplexy is rare, occurring in 0.2 per 100 patient years among untreated patients [17]. Prior work on the risk factors for pituitary apoplexy has been primarily limited to small retrospective series [18] but has generally found that large tumors, male gender, rapidly growing tumors, non-functional tumor status, and tumors that invade the cavernous sinus are risk factors for apoplexy [4,17]. The role of anti-thrombotic therapies in apoplexy also remains controversial, with some authors reporting an association between anticoagulation and subsequent apoplexy [17] while others report no such risk [19]. Herein, we demonstrated no cases of pituitary apoplexy following IVT for acute ischemic stroke.

Our study has several limitations. First, ascertainment of pituitary adenomas may be suboptimal. However, given that the population prevalence of pituitary adenomas (100 per 100,000 persons, or roughly 0.1%) closely mirrors the prevalence observed in our study (0.13%), the risk of under-ascertainment is likely low. Second, given that computed tomography is the primary imaging modality for acute stroke triage at most institutions in the United States, it is possible that some pituitary masses, particularly microadenomas, may not be identified at the time of IVT decision-making and are instead diagnosed on follow-up magnetic resonance imaging. Nevertheless, our findings that IVT is safe and effective should not be influenced by the time of pituitary adenoma detection. Another limitation of this study is the inability to distinguish between microadenomas, macroadenomas, and other sellar lesions such as craniopharyngiomas, which have varying risks of pituitary apoplexy [4]. Additionally, patients who received endovascular therapy [20] were excluded from this analysis to better isolate the therapeutic benefits and hemorrhagic risks of IVT without the influence of other reperfusion treatments. Thus, our study population is inherently biased towards patients with more minor and distal strokes, though we do not expect that stroke size or location would moderate the risks of IVT attributed to the presence of pituitary neoplasms. While we attempted to control for potential confounders, the retrospective nature of this work has potential unmeasured confounders that could bias the results, including tumor size, radiographic characteristics, endocrinopathy status, and clinician risk tolerance. Finally, while discharge destinations are reasonable surrogates for clinical outcomes, they are indirect measures of neurological function. Dedicated prospective registry studies with longer-term follow-up are needed to confirm our findings.

## 5. Conclusions

Only five cases of IVT for AIS patients with pituitary neoplasia were identified during a scoping review of the current literature, highlighting a striking lack of clinical data. In a nationwide cohort study in the United States, we found that for AIS patients with concomitant pituitary neoplasia, IVT was associated with improved hospitalization outcomes without increasing the risk of intracranial hemorrhage or pituitary apoplexy.

## Figures and Tables

**Table 1:** Systematic review of patients with pituitary neoplasia who received IVT for acute ischemic stroke [11,12].

Study	Setting	Number of Patients with Sellar Neoplasia	Number of Intracranial Hemorrhages	Number of Symptomatic Intracranial Hemorrhages	Number of Apoplexies
Evans et al. (2024) [12]	Single center, Great Britain	1	0 (0%)	0 (0%)	0 (0%)
Seystahl et al. (2023) [11]	Multicenter registry, Europe	4 (1 suspected craniopharyngioma)	3 (75%)	2 (50%)	0 (0%)
Total		5	3 (60%)	2 (40%)	0 (0%)

**Table 2:** Patient characteristics.

Characteristic—% (n) or Mean (SD)	No Pituitary Neoplasm *N* = 1,245,089	Pituitary Neoplasm *N* = 1661	*p*-Value
Intravenous thrombolysis	17.3% (215,450)	14.4% (240)	0.017
Age (years)	71 (60–81)	72 (60–81)	0.88
Female sex	48.4% (602,559)	43.7% (726)	0.003
Teaching hospital	76.8% (955,987)	80.6% (1339)	0.01
NIH stroke scale	4 (2–8)	4 (2–7)	0.002
Chronic medications			
Anticoagulant	12.4% (154,586)	7.9% (131)	<0.001
Antiplatelet	11.5% (143,783)	12.1% (202)	0.59
Comorbidities			
Hypertension	87.2% (1,085,832)	88.4% (1469)	0.32
Atrial fibrillation	23.1% (287,201)	17.2% (286)	<0.001
Diabetes	39.8% (496,050)	42.2% (702)	0.17
Smoking	37.9% (471,482)	31.6% (525)	<0.001
Hyperlipidemia	65.0% (809,316)	69.8% (1160)	0.002
Dementia	11.1% (138,645)	10.6% (177)	0.67
Cervical artery dissection	1.1% (13,688)	1.0% (17)	0.87
Intracranial atherosclerosis	4.4% (55,240)	6.0% (100)	0.018
Peripheral artery disease	2.4% (29,299)	2.7% (45)	0.45
Myocardial infarction	2.9% (36,560)	1.7% (29)	0.031
Ischemic heart disease	25.1% (312,207)	22.5% (373)	0.071
Chronic kidney disease	19.0% (236,397)	18.7% (310)	0.79
Liver disease	2.1% (26,502)	2.7% (44)	0.3
Congestive heart failure	18.7% (233,277)	14.2% (236)	<0.001
Pulmonary embolism	1.9% (23,882)	2.0% (34)	0.83
Mood disorder	14.8% (184,327)	16.3% (271)	0.19
Anxiety disorder	13.6% (169,768)	12.5% (208)	0.46
Headache disorder	4.4% (54,846)	6.2% (103)	0.053
Coagulopathy	5.3% (65,727)	3.7% (61)	0.034
Elixhauser comorbidity index	11 (6–17)	9 (5–15)	<0.001

**Table 3:** Study outcomes. Values < 10 are shown as such per HCUP guidelines.

Outcomes—% (n) or OR [95%CI]	Intracranial Hemorrhage	Routine Discharge	Home Discharge	Death
Patients with pituitary mass				
IVT (*n* = 240)	<4.6% (<11)	46.8% (112)	70.9% (170)	<4.6% (<11)
No IVT (*n* = 1421)	4.0% (58)	37.0% (526)	58.9% (837)	1.3% (18)
IVT vs. no IVT				
Adjusted OR [95%CI]	0.77 [0.35–1.72]	2.46 [1.56–3.87]	2.63 [1.55–4.45]	1.72 [0.49–6.04]
*p*-value	0.53	<0.001	<0.001	0.40
Patients without pituitary mass				
IVT (*n* = 215,450)	8.7% (18,699)	47.8% (102,956)	66.8% (143,830)	4.1% (8847)
No IVT (*n* = 1,029,639)	4.8% (49,152)	37.0% (381,198)	59.2% (609,525)	3.6% (37,451)
IVT vs. no IVT				
Adjusted OR [95%CI]	1.71 [1.65–1.78]	2.07 [2.02–2.12]	1.82 [1.78–1.85]	0.90 [0.87–0.94]
*p*-value	<0.001	<0.001	<0.001	<0.001
Inter-group comparisons				
Mass vs. no mass among IVT-treated patients				
Adjusted OR [95%CI]	0.38 [0.15–1.02]	0.89 [0.51–1.57]	1.41 [0.77–2.59]	0.66 [0.21–2.03]
*p*-value	0.054	0.69	0.26	0.47
Interaction *p*-value of pituitary mass vs. IVT	0.063	0.73	0.19	0.29

## Data Availability

Data can be made available upon reasonable written request.

## Supplementary Materials

The following supporting information can be downloaded at https://www.mdpi.com/article/10.3390/neurosci7010019/s1, Table S1: Search terms for systematic review; Table S2: ICD-10 Codes utilized for this study.
