# Supplementary material for: Intravenous Thrombolysis for Ischemic Stroke Patients with Pituitary Neoplasms: A Nationwide Study and Scoping Review
Source: NeuroSci. 2026 Feb 2;7(1):19. doi: 10.3390/neurosci7010019 (PMC12922147; doi:10.3390/neurosci7010019)
Supplement: Supplementary file 1 [file neurosci-07-00019-s001.zip › neurosci-4002493-supplementary.pdf]

NeuroSci

MDPI

Intravenous thrombolysis for ischemic stroke patients with pituitary neoplasms: a nationwide study and scoping review

Matthew K. McIntyre, MD[1^], Huanwen Chen, MD[2,3^], Dheeraj Gandhi, MD[3], Ajay Malhotra, MD[4], Ryan Priest, MD[5], Marco Colasurdo, MD[5*]

[1]Department of Neurological Surgery, Oregon Health and Science University, Portland OR
[2]Department of Neurology, MedStar Georgetown University Hospital, Washington, DC 20007, USA
[3]Department of Neurosurgery, University of Maryland Medical Center, Baltimore, MD 21201, USA
[4]Department of Radiology, Yale New Haven Hospital, New Haven, CT 06510, USA
[5]Department of Interventional Radiology, Oregon Health & Science University, Portland, OR 97239, USA

Supplemental Data

Database	Search Terms
**PubMed**	(stroke[MeSH Terms] OR stroke[Title/Abstract] OR infarct*[Title/Abstract] OR ischemia[Title/Abstract] OR ischaemia[Title/Abstract] OR "cerebral infarction"[MeSH Terms]) AND (thrombolysis[Title/Abstract] OR "thrombolytic therapy"[MeSH Terms] OR alteplase[Title/Abstract] OR tenecteplase[Title/Abstract] OR thrombolytic*[Title/Abstract] OR tPA[Title/Abstract] OR "tissue plasminogen activator"[Title/Abstract] OR urokinase[Title/Abstract] OR "fibrinolytic agents"[MeSH Terms]) AND ("pituitary*"[Title/Abstract] OR apoplexy*[Title/Abstract] OR "sella*"[Title/Abstract] OR "cavernous hemangioma*"[Title/Abstract] OR "Pituitary Neoplasms"[MeSH Terms])
**Embase (Elsevier)**	('cerebrovascular accident'/exp OR stroke:ab,ti OR infarct*:ab,ti OR ischemia:ab,ti OR ischaemia:ab,ti OR 'brain infarction'/exp) AND ('fibrinolytic therapy'/exp OR thrombolysis:ab,ti OR 'alteplase'/exp OR alteplase:ab,ti OR 'tenecteplase'/exp OR tenecteplase:ab,ti OR thrombolytic*:ab,ti OR tpa:ab,ti OR 'tissue plasminogen activator':ab,ti OR 'urokinase'/exp OR urokinase:ab,ti) AND ('pituitary'/exp OR 'apoplexy*':ab,ti OR sella*:ab,ti)
**Web of Science**	TS=((stroke OR infarct* OR ischemia OR ischaemia OR "cerebral infarction") AND (thrombolysis OR alteplase OR tenecteplase OR thrombolytic* OR tPA OR "tissue plasminogen activator" OR urokinase OR "fibrinolytic therapy") AND ("pituitary*" OR “apoplexy*” OR "sella*"))
**Scopus**	TITLE-ABS-KEY((stroke OR infarct* OR ischemia OR ischaemia OR "cerebral infarction") AND (thrombolysis OR alteplase OR tenecteplase OR thrombolytic* OR tPA OR "tissue plasminogen activator" OR urokinase OR "fibrinolytic therapy") AND ("pituitary*" OR “sella*” OR "apoplexy*"))

Supplemental Table S1: Search terms for systematic review.

Variable	Code(s)
**Inclusion**	
Stroke (top dx code)	G463, G464, G465, G466, G467, I6300, I63011, I63012, I63013, I63019, I6302, I63031, I63032, I63033, I63039, I6309, I6310, I63111, I63112, I63113, I63119, I6312, I63131, I63132, I63133, I63139, I6319, I6320, I63211, I63212, I63213, I63219, I6322, I63231, I63232, I63233, I63239, I6329, I6330, I63311, I63312, I63313, I63319, I63321, I63322, I63323, I63329, I63331, I63332, I63333, I63339, I63341, I63342, I63343, I63349, I6339, I6340, I63411, I63412, I63413, I63419, I63421, I63422, I63423, I63429, I63431, I63432, I63433, I63439, I63441, I63442, I63443, I63449, I6349, I6350, I63511, I63512, I63513, I63519, I63521, I63522, I63523, I63529, I63531, I63532, I63533, I63539, I63541, I63542, I63543, I63549, I6359, I636, I6381, I6389, I639
NIHSS 1 or greater	R297 except R29.70
IVT	3E03317,Z9282
Pituitary mass	D352, C751
Apoplexy	E236
**Exclusion**	
Vasculitis/arteritis	I677, M31
Endocarditis	I33
Cerebral amyloid angiopathy	I680
Moyamoya disease	I675
Intracranial mass	C70, C71, D32, D330, D331, D333, D332, C7931, C7932
Cerebrovascular malformation	Q282,Q283,I671
Endovascular thrombectomy	03CG3, 03CK3, 03CL3, 03CP3, 03CQ3
Endovascular angioplasty	037G3 ,037H3, 037J3, 037K3, 037L3, 037P3, 037Q3
**Comorbidities**	
Afib	I480,I4811,I4819,I4820,I4821,I483,I484,I4891,I4892
Chronic kidney disease	N18
Ischemic heart disease	I25, I21, I22
Peripheral artery disease	I70, K550, K551
Dementia	F00. F01. F02. F03. G30, I6991
Intracranial atherosclerosis	I672
Hyperlipidemia	E7800, E7801, E781,E782,E783,E7841,E7849,E785
Smoking	Z720,Z87891,F17210
Anticoagulant use	D6832,Z7901
Antiplatelet use	Z7902
Chronic kidney disease	N18
Headache disorder	G44, G43, R51
Dissection	I7771,I7774,I7775
Anxiety disorder	F40, F41, F42, F43, F44, F45, F48

Mood disorders	F30, F31, F32, F33, F34, F39,

All other comorbidites within Elixhauser and Charleston comorbidit indices are extraced using "icd" package (v 4.0.9) in R.

Supplemental Table S2: ICD-10 Codes utilized for this study.
